# Burden of herpes zoster-associated chronic pain in Italian patients aged 50 years and over (2009–2010): a GP-based prospective cohort study

**DOI:** 10.1186/s12879-014-0637-6

**Published:** 2014-12-06

**Authors:** Hélène Bricout, Emilia Perinetti, Paolo Marchettini, Pietro Ragni, Carla Maria Zotti, Giovanni Gabutti, Antonio Volpi, Elisabetta Franco

**Affiliations:** Epidemiological Department, Sanofi Pasteur MSD, Lyon, France; Medical & Scientific Department, Sanofi Pasteur MSD, Rome, Italy; Pain Medicine Center, Hospital San Raffaele of Milan, Milan, Italy; Local Health Authority for Reggio Emilia, Emilia Romagna, Italy; Department of Public Health and Paediatrics, University of Turin, Turin, Italy; Department of Medical Sciences, University of Ferrara, Ferrara, Italy; Department of Clinical Sciences, University of Rome Tor Vergata, Rome, Italy; Department of Biomedicine and Prevention, University of Rome Tor Vergata, Rome, Italy; Pain Pathophysiology and Therapy, University School of Italian Switzerland, Manno Lugano, Switzerland

**Keywords:** Post herpetic neuralgia, Herpes zoster, Shingles, Pain, Varicella zoster virus, Vaccine-preventable diseases, Prospective study, Italy

## Abstract

**Background:**

Post-herpetic neuralgia (PHN) is the most common complication in herpes zoster (HZ) patients.

**Methods:**

We performed a longitudinal, prospective study in 108 general practices throughout Italy to assess how many immunocompetent patients aged ≥50 years with newly diagnosed HZ develop HZ-associated pain, its duration and management over 6-months. HZ-associated pain was assessed by a direct question to the patient and by self-assessment of the worst pain felt in the previous two weeks on a visual analogue scale (VAS), a score ≥3 was taken as pain. PHN was defined as pain reported during the study period persisting for ≥3 months. Quality of life (QoL) was measured using the SF-12 questionnaire.

**Results:**

At enrolment, 370 of the 413 patients (89.6%) reported HZ-associated pain which was still present in 20.6% and 9.2% of patients after three and six months, respectively, despite many patients receiving recommended anti-viral therapy. The overall QoL scores were lower than those in healthy Italians of similar age; scores for patients with HZ-associated pain were lower. The presence of >50 vesicles and VAS score ≥3 at enrolment, and being male were significantly associated with PHN at three months.

**Conclusions:**

These results suggest that HZ and PHN represent an important burden of disease in the elderly. There is a need for interventions that can prevent and reduce the burden of HZ to help improve the quality of life of the elderly. These data may be useful as baseline epidemiology data for the assessment of the impact of the VZV vaccine in Italy, after its implementation.

**Electronic supplementary material:**

The online version of this article (doi:10.1186/s12879-014-0637-6) contains supplementary material, which is available to authorized users.

## Background

Varicella zoster virus (VZV) is a herpes virus that infects nearly all humans in temperate regions resulting in a highly contagious primary infection of varicella, usually in childhood [1]. After this infection, the virus remains latent in sensory ganglia until it is reactivated to cause herpes zoster (HZ) [2]. In Europe, almost all adults (>95%) are healthy carriers of VZV following childhood varicella and are, therefore, at risk of developing HZ [3].

The immune system declines with age (immunosenescence); the decline of VZV-specific T-cell-mediated immunity is thought to be involved in this reactivation [4]. The incidence and severity (number of vesicles, and pain intensity and duration) of HZ increase with age, but it is difficult to predict who will develop HZ [5],[6]. Although patients with treatment- or disease-associated immunosuppression are at higher risk for HZ, 90% of patients with HZ are immunocompetent. Other risk factors include sex, ethnicity, genetic susceptibility, familial history of HZ, mechanical trauma, psychological stress, depression and diabetes [7]-[9].

Herpes zoster is characterized by a, usually painful, unilateral vesicular rash, generally limited to a single dermatome, corresponding to the sensory ganglion from which the latent VZV was reactivated [10]. The most frequent localization (>50%) is thoracic, followed by cranial, including the ophthalmic division of the trigeminal nerve; the incidence of this latter localization increases with age [11].

Pain may occur during three distinct phases of the disease:

prodromal pain: often occurs prior to rash onset;

acute pain or neuritis: begins with the rash and usually disappears within 2 to 4 weeks;

chronic pain i.e. post-herpetic neuralgia (PHN): most commonly defined as pain occurring or persisting for ≥3 months after rash onset.

HZ-associated pain is debilitating and often has a substantial, negative impact on patients’ normal life and health, particularly among the elderly whose activities and basic tasks of daily living can be impaired and anxiety and depression can occur [12],[13]. In addition, when PHN occurs, pain management is difficult and patients suffer from important side effects [14]. Treatment with antiviral agents within 72 hours of rash onset for seven days is recommended in some patient groups to accelerate rash healing and to limit the severity and duration of pain [15]. However, observational studies in France, Italy, the Netherlands and the UK and a meta-analysis gave heterogeneous results [16]-[20].

The annual HZ incidence is similar throughout Europe, increasing with age from around 1–4/1 000 in adults aged <50 years to about 7–8/1 000 in those ≥50 years, up to 10/1 000 at >80 years [21]. After the introduction of antivirals drugs, about 10% to 30% of patients aged ≥50 years with HZ develop PHN in Europe [22]. Most studies estimating the percentage of patients with HZ who develop PHN are retrospective. In addition, there is no consensus for the definition of PHN making comparisons between studies difficult [22].

In Italy, there are insufficient data on the burden of HZ and PHN because there is no HZ-specific national surveillance system. In one nationwide study the annual incidence of general practitioner (GP) visits for HZ was 4.1/1 000 persons aged ≥15 years; 19.6% presented with PHN and 26.1% presented ≥1 complications [23]. One study in 2004 in the Piedmont region reported an overall HZ incidence rate of 1.59/1 000 inhabitants and a hospitalization incidence rate of 0.12/1 000 [24]. In another study that analysed data from four regions in Italy, the HZ incidence in adults aged ≥50 years was 6.6/1 000, and 9.4% and 7.2% of the patients had PHN at 1 month and 3 month, respectively [25]. Another study that analysed data from 1999 to 2005 reported a mean annual number of 4 503 HZ hospitalisations and 543 day-hospitalisations; 62% of patients were >65 years [26].

In the perspective of a VZV vaccine to prevent and reduce the severity of HZ becoming available in Europe, it was decided to obtain baseline data on the occurrence of PHN in a multicenter, longitudinal, prospective, observational study involving general practitioners (GPs) throughout Italy, the HER.O.E.S. Study – HERpes zoster Outcome: Epidemiological Study. The primary objective was to assess the percentage of patients with HZ that have HZ-associated chronic pain and its duration.

## Methods

This study was conducted in compliance with the Declaration of Helsinki (Seoul revision; 2008), Good Epidemiological Practice Guidelines and Italian national regulations [27],[28]. The protocol and study documentation were approved by Ethics Committee for each local health unit (See Table 1).

**Table 1:** **List of local ethics committees that approved the protocol and study documentation**

Local health unit	IRB/IEC name	IRB/IEC address
Alessandria	Comitato Etico	Via Venezia, 16
	Azienda Osped. SS. Antonio e Biagio e Cesare Arrigo	15100 Alessandria
Asolo	Comitato Etico	Borgo Cavalli, 42
	Azienda ULSS N. 9 di	31100 Treviso
	Treviso	
Asti	Comitato Etico	Via Venezia, 16
	Azienda Ospedaliera SS. Antonio e Biagio	15100 Alessandria
	e Cesare Arrigo	
Bologna	Comitato Etico	Via Gramsci, 12
	Azienda USL di Bologna	40121 Bologna
	Dipartimento Farmaceutico	
Brindisi	COMITATO ETICO	Via Napoli, 8
	ASL n. 1 di BRINDISI	72100 Brindisi
Bussolengo	Comitato etico	Via Salvo D’Acquisto, 7
	Provincia di Verona Azienda ULSS 20 di Verona c/o Dipartimento	37122 Verona
	Farmaceutico	
Carbonia	Comitato Etico	Via Dalmazia, 83
	ASL di Carbonia	09013 Carbonia
Catanzaro	Comitato Etico	Via Vinicio Cortese, 25
	Azienda Sanitaria	88100 Catanzaro
	Provinciale di Catanzaro	
Ferrara	Comitato Etico	Corso Giovecca, 203
	c/o Ufficio Ricerche e Innovazioni	44100 Ferrara
	Azienda Ospedaliero, Universitaria di Ferrara	
Genova	Comitato Etico	Via Bertani, 4
	Asl 3 Genovese	16125 Genova
Grosseto	Comitato Etico	Via Genova, 6/D
	c/o U.O. Farmaceutica Territoriale - Azienda USL n. 9 di Grosseto	58100 Grosseto
Lecce	Comitato Etico	Via Miglietta, 5
	ASL di Lecce	73100 Lecce
Milano	Comitato Etico ASL Città di Milano	Via Statuto, 5
	c/o Servizio Farmaceutico	20121 Milano
Napoli/5	Comitato Etico	Piazza San Giovanni
	ASL Napoli 3 Sud	80031 Brusciano (NA)
Pavia	Comitato Etico	Via Indipendenza, 3
	Asl Provincia di Pavia	27100 Pavia
Pescara	Comitato Etico	Via Fonte Romana, 8
	ASL di Pescara	65100 Pescara
Ragusa	Comitato Etico	Piazza Igea, 2
	Azienda USL 7 di Ragusa	97100 Ragusa
Roma/C	Comitato Etico	Piazzale Umanesimo, 10
	c/o Direzione Amministrativa Ospedale Sant’Eugenio	00144 Roma
Roma/H	Comitato Etico	Borgo Garibaldi, 12
	ASL Roma H di Albano	00041 Albano Laziale (RM)
	Laziale	
Teramo	Comitato Etico	Circonvallazione Ragusa, 1
	c/o Servizio Farmaceutico Territoriale - ASL 106 di Teramo	64100 Teramo
Torino/1	Segreteria Tecnico-Scientifica del Comitato Etico	Corso Svizzera 185 BIS
	ASL TO 2	10149 Torino
Treviso	Comitato Etico	Borgo Cavalli, 42
	Azienda ULSS N. 9 di	31100 Treviso
	Treviso	
Varese	Segreteria del Comitato Etico c/o Direzione Sanitaria ASL della	Via Ottorini Rossi, 9
	Provincia di Varese	21100 Varese
Verona	Comitato Etico	Via Salvo D’Acquisto, 7
	Provincia di Verona Azienda ULSS 20 di Verona c/o Dipartimento Farmaceutico	37122 Verona
Vibo Valentia	Comitato Etico	Via Dante Alighieri, Palazzo
	ASL 8 di Vibo Valentia	ex
		Inam - 89900 Vibo Valentia

Note: sites of Genova, Pavia, Alessandria, Asolo were not activated and did not enrol patients.

### Study objectives

The co-primary objectives of the six-month study were:

to assess the percentage of patients with HZ who developed HZ-associated chronic pain and how long the pain lasted.

to collect disease management data from diagnosis to resolution of the acute symptoms, and data on the management of HZ-related pain.

The secondary objectives were to assess quality of life (QoL) in the patients with HZ/PHN using the SF-12 instrument and to assess HZ-related pain intensity using a visual analogue scale (VAS). We also recorded the presence of clinical variables that have been identified as risk factors for HZ and assessed the patients’ care pathway and their disease progression during the study period.

### Study design

Patients aged >50 years old with a new clinical diagnosis of HZ, who were immunocompetent and who signed an informed consent form were enrolled in the study. Each patient was expected to attend four visits at 0, 1 (± 15 days), 3 (± 21 days) and 6 (± 30 days) months. Patients could be excluded from the analysis for one visit for non-respect of the time window, but included in the analysis for a subsequent visit, if it occurred within the time window.

At the initial visit (V0), descriptive information was collected: socio demographic data, clinical description, medical history. The patients were asked if they had HZ-associated pain at the visit and their quality of life was assessed using the physical component summary (PCS-12) and the mental component summary (MCS-12) scores from the SF-12 questionnaire. The scores ranged from 0 (lowest level of health) to 100 (highest level of health) [29]. A score of <50 indicates a below-average health status. The level of pain was assessed using a visual analogue scale (VAS) for the worst pain felt during the previous two weeks, with scores ranging from 0 (‘no pain’) to 10 (‘worst pain imaginable’). A VAS score of ≥3 was considered to be correlated with pain interfering with activities of daily living [30]. At each follow-up visit the presence of HZ-associated pain, the level of pain using the VAS and quality of life (QoL) scales were assessed. In addition, data on the clinical evolution and the health care resources used were collected.

The presence of allodynia, itching, paresthesia and malaise were all assessed by the physicians during the clinical examination at corresponding study visit. Data were collected using an electronic case report form (eCRF) which enabled a real-time quality control of the data at entry. In addition, a clinical research assistant telephoned the GPs monthly and also performed *ad hoc* monitoring visits during the study.

### Assessment of HZ-associated pain

In HEROES, PHN (primary outcome) was defined as pain that persisted for more than three months after rash onset assessed by the patient replying ‘yes’ at a study visit when asked by the GP about the presence of HZ-associated pain. This assessment mode was chosen to be pragmatic, since physicians do not use tools to measure pain systematically. For the secondary outcome, HZ-associated pain was assessed with a score ≥3 on the VAS, reflecting the worst pain the patient had felt during the two-weeks prior to the visit.

### Sample size calculation

Based on information obtained from a questionnaire survey conducted on 317 GPs throughout Italy in 2008 that collected data on the number of patients with HZ they treated annually and how many had PHN, and other published data, [31]-[33] we assumed that between 15% and 40% of patients with HZ would have HZ-associated pain. Thus, with a threshold of p = 0.05 and a pain incidence of 40%, it was estimated that we needed to enrol 400 patients to guarantee a precision of ±4.8%. We decided to enrol 500 patients to allow for 20% erroneously-enrolled patients.

### Statistical analyses

The descriptive analyses were performed with SAS (Statistical Analysis System), V9.2. The recorded or derived variables were summarized as frequencies (absolute or relative) or means or medians with their associated distribution (number of observations, mean, standard deviation, median, minimum, maximum). For all clinically-relevant variables, 95% confidence intervals (CIs) were calculated. Analyses were stratified on gender, age group (50–59, 60–69 and ≥70), and presence of pain at the respective visit.

Variables present at the initial visit associated (p = 0.25) with PHN at 3-months in univariate analyses were included in a multivariate regression model to identify PHN predictive factors (p ≤ 0.05). The fit of the final model was assessed using the Hosmer and Lemeshow goodness-of-fit test. The estimated β coefficients and the corresponding 95% CIs were calculated. Odds ratios and the corresponding 95% CIs were also calculated.

## Results

### Patient population

**Figure 1:**
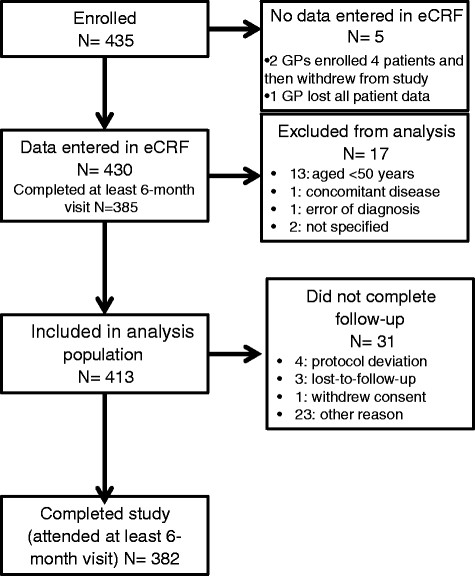
**Disposition of included patients.**

A total of 108 of the 147 GPs who accepted to participate in the study included at least one patient. These 108 GPs were from 21 local health units localized in 12 Italian regions. Their regional distribution was similar to the national distribution (data not shown). They enrolled 435 patients with a new diagnosis of HZ from 27 March 2009 to 5 July 2010 of whom 413 were included in the study (Figure 1).The majority of the GPs (72.2%) respected the time windows for all their patients’ follow-up visits.

The patients were aged 67.9 ± 10.7 years old and 64.2% were women (Table 2). Almost half (46.7%) of the study population were aged ≥70 years. The majority (81.4%) lived with their family; about 25% were working. Most patients (90.3%) had been followed by their GP for >5 years with a mean of 8.2 visits during the previous 12 months. More than 60% of the patients presented ≥1 chronic condition (diabetes, chronic cardiovascular disease or chronic respiratory diseases); the percentage increased with age.

**Table 2:** **Baseline characteristics of included subjects**

Number of patients (%)	50-59	60-69	≥70	Males	Females	Total
	107 (25.9)	113 (27.4)	193 (46.7)	148 (35.8)	265 (64.2)	413 (100)
**Age, years, mean ± SD**	54.5 ± 2.8	64.4 ± 2.9	77.4 ± 6.2	68.3 ± 11.0	67.7 ± 10.6	67.9 ± 10.7
**Gender, %, M/F**	33.6/66.4	33.6/66.4	38.3/61.7	-	-	35.8/64.2
**Employment status n (%)**						
Worker	79 (73.8)	23 (20.4)	2 (1.0)	46 (31.1)	58 (21.9)	104 (25.2)
Retired	11 (10.8)	76 (67.3)	184 (95.3)	102 (68.9)	169 (63.8)	271 (65.6)
Other	17 (15.9)	14 (12.4)	7 (3.6)	-	38 (14.3)	38 (9.2)
**Medical history, n (%)**						
Diabetes	14 (13.1)	19 (16.8)	39 (20.2)	25 (16.9)	47 (17.7)	72 (17.4)
Type 1	3 (2.8)	0	3 (1.6)	4 (2.7)	2 (0.8)	6 (1.5)
Type 2	11 (10.3)	19 (16.8)	36 (18.7)	21 (12.2)	45 (17.0)	66 (16.0)
Chronic cardiovascular diseases	26 (24.3)	61 (54.0)	139 (72.0)	84 (56.8)	142 (53.6)	226 (54.7)
Chronic respiratory diseases	6 (5.6)	13 (11.5)	33 (17.1)	27 (18.2)	25 (9.4)	52 (12.6)
Other relevant diseases	29 (27.1)	51 (45.1)	100 (51.8)	60 (40.5)	120 (45.3)	180 (43.6)
**Rash localization n (%)**						
Cranial	16 (15.0)	14(12.4)	22 (11.4)	15 (10.1)	37 (14.0)	52 (12.6)
Cervical	19 (17.8)	23 (20.35)	20 (10.4)	19 (12.8)	43 (16.2)	62 (15.01)
Thoracic	61 (57.0)	63 (55.8)	120 (62.2)	92 (62.2)	152 (57.4)	244 (59.1)
Lumbar	15 (14.0)	12 (10.62)	30 (15.5)	22 (14.9)	35 (13.2)	57 (13.8)
Sacral	12 (11.2)	11 (9.73)	23 (11.9)	17 (11.5)	29 (10.9)	46 (11.1)
**Number of vesicles, n (%)**						
None	5 (4.7)	4 (3.5)	6 (3.1)	6 (4.1)	9 (3.4)	15 (3.6)
Less than 50	83 (77.6)	88 (77.9)	147 (76.2)	112 (75.7)	206 (77.7)	318 (77.0)
More than 50	18 (16.8)	21 (18.6)	40 (20.7)	30 (20.3)	49 (18.5)	79 (19.1)
**SF-12 (mean, SD)**						
Physical Health Score (PCS-12)	42.7 ± 7.6	41.4 ± 8.0	37.6 ± 9.2	41.5 ± 8.4	39.4 ± 8.8	40.1 ± 8.7
Mental Health Score (MCS-12)	41.9 ± 10.8	42.6 ± 10.0	41.0 ± 10.9	45.2 ± 10,0	39.8 ± 10.4	41.7 ± 10.6

### Clinical characteristics and treatment of HZ

The mean delay from HZ prodrome and rash onset to consultation was 4.5 days and 3 days, respectively. This delay was longer in older patients; delay since prodrome: 3.7 days for those aged 50–59; 4.3 days for those aged 60–69 and 5.1 days for those aged ≥70 and since rash onset: 2.3 days for those aged 50–59; 2.7 days for those aged 60–69 and 3.2 for those aged ≥70. All patients (100%) presented with a rash and 77% had <50 vesicles. The rash was mainly localized in the thoracic dermatome area (59.1%) followed by cervical (15.0%), lumbar (13.8%), cranial (12.6%) and sacral segment (11.1%) dermatome areas, respectively (Table 2).

Among the patients presenting with pain at the initial visit, 59.7% had allodynia, 74.3% itching and 58.9% paresthesiae (Table 3). Many patients who reported pain during the study follow-up visits still had allodynia (49.2%, 56.2% and 52.9% at the 1-month, 3-month and 6-month visits, respectively) whereas there was a decrease in the percentage of patients with paresthesiae and itching.

**Table 3:** **Assessment and characteristics of the HZ-associated pain and quality of life scores at each study visit**

	Visit			
	Enrolment	1 month	3 months	6 months
**Number of patients who completed the visit**	413	390	353	368
**Patients reporting pain at the respective visit**				
**Number of patients**	**370**	**193**	**73**	**34**
Mean VAS score for patients reporting pain ± SD	5.8 ± 2.4	4.4 ± 2.3	3.7 ± 2.0	3.7 ± 2.3
**Main characteristics of HZ-associated pain (%)**				
Allodynia	59.7	49.2	56.2	52.9
Itching	74.3	27.5	21.9	17.7
Paresthesia	58.9	49.7	52.1	38.2
Malaise	20.8	14.0	13.7	8.8
**Quality of life score (SF12)**				
Mean PCS-12 score ± SD	39.2 ± 8.4	37.7 ± 8.3	40.1 ± 8.4	39.0 ± 9.3
Mean MCS-12 score ± SD	41.6 ± 10.4	39.3 ± 10.4	39.6 ± 9.8	39.4 ± 10.6
**Patients with a VAS score ≥3 at the respective visit**				
**Number of patients**	308	175	71	50
Mean VAS score among these patients	6.4 ± 1.9	5.2 ± 1.9	4.9 ± 1.8	5.4 ± 2.1

Most of the patients (91.5%) received an oral antiviral (acyclovir: n = 149; brivudin: n = 103; valacyclovir: n = 86; famciclovir: n = 42) for a median duration of 6.0 days. Oral antiviral treatment was initiated a median of two days after rash onset (mean = 3.4 days); 70% of the patients initiated oral antiviral treatment within three days after rash onset. Topical antiviral treatment was administered to 9.4% of patients (acyclovir: n = 37; tromantadine hydrochloride: n = 2).

Only 23% of the patients received pain therapy for a median duration of 12.5 days (mean = 3 days); analgesics (codeine-paracetamol, oxycodone, paracetamol, tramadol, tramadol-paracetamol) (n = 48), nonsteroidal anti-inflammatory drugs (NSAID)s (n = 36), anti-epileptics (n = 29) or psycho-analeptics (n = 12).

Among the 104 patients who worked at the initial visit, 36 took sick-leave due to their HZ episode for: 1 to 5 days (53%); 6 to 10 (33%); and more than 10 days (14%). At the 1-month visit, 33 patients reported that they had taken sick-leave, 2 at the 3-month visit and 1 at the 6-month visit.

About 12% of the patients (n = 52) consulted ≥1 specialist during the study (mainly a dermatologist, neurologist or ophthalmologist). None of the patients were hospitalised due to HZ.

### Herpes zoster-associated pain

**Figure 2:**
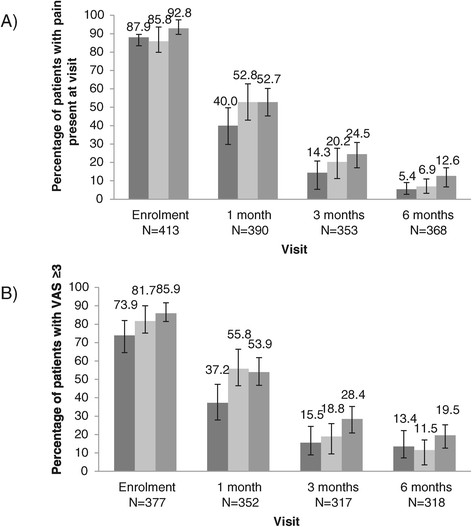
**Percentage of patients with HZ-associated pain by age group (50–59; 60–69 and ≥70) and by visit A) presence of pain at visit and B) pain in the previous two weeks with a VAS score ≥3.**

**Figure 3:**
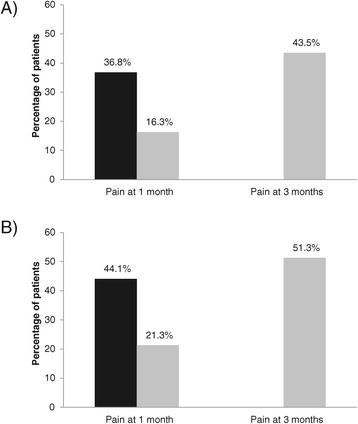
**Percentage of patients with PHN (reported pain at the visit) at 3 (black bar) and 6 months (grey bar) among patients with pain at 1 month and 3 months. A**: all patients aged ≥50; **B**: patients aged ≥ 70. 36.8% of patients aged ≥ 50 years and 44.1% of those aged ≥70 years with pain at 1 month had persistent pain at 3 months. 43.5% of patients aged ≥50 years and 51.3% of those aged ≥70 years with persistent pain at 3 months still had pain present at 6 months.

Overall, 370/413 (89.6% [86.2-92.3]) patients reported HZ-associated pain at the initial visit with a mean score of 5.8 ± 2.4 (Table 3). Among the 353 patients who completed the three-month visit 73 (20.6% [16.5-25.2]) still reported pain, satisfying our definition for PHN; the percentage increased with age (14.3% of those aged 50–59 years, 20.2% of those aged 60–69 years and 24.5% of those aged ≥70 years) (Figure 2A). PHN was still present in 34 of the 368 patients at the six-month visit (9.2% [6.5-12.7]). The prevalence of pain at the month 3 and 6 visits was higher among patients who reported pain at the one-month visit (Figure 3). There was a non-statistically significant trend for more men than women to report pain at the one- and three-month visits (all p values >0.05).

The mean VAS score for patients who reported pain at each visit decreased from 5.8 (median 6) at enrolment to 3.7 (median 3) at the six-month visit. The percentage of patients with a VAS score ≥3 was 81.7% [77.6% - 85.4%] at the initial visit; 49.7% [44.5% - 54.9%] at the 1-month visit; 22.4% [18.1% - 27.2%] at the 3-month visit and 15.2% [12.0% - 20.0%] at the 6-month visit (Figure 2B).

### Predictive factors for persistent HZ-associated pain

The results from the univariate analyses showed that the presence at the initial visit of >50 vesicles, allodynia, a VAS score ≥3, the PCS-12 score, the presence of diabetes or chronic cardiovascular disease or respiratory disease, age, a rash localized on the lumbar dermatome, being male and the MCS-12 score were potential predictive factors for the persistence of pain at three months (PHN). Multivariate analysis, including these variables, showed that the presence of >50 vesicles (OR = 2.595 [1.267-5.314]), a VAS score ≥3 (OR = 4.599 [1.181-17.907]) and being male (OR = 2.017 [1.006-4.042]) were significantly associated with PHN (p < 0.05) (Table 4).

**Table 4:** Predictive factors for persistentHZ-associated pain – results from multivariate regression

Explanatory variable	Level of explanatory variable	P value	OR estimates	95% CI
More than 50 vesicles	Yes vs no	0.0091	2.595	1.267-5.314
VAS score ≥3 at V0	Yes vs no	0.0278	4.599	1.181-17.907
Gender	Male vs female	0.0481	2.017	1.006-4.042
Presence of allodynia	Yes vs no	0.1039	1.812	0.005-3.710
MCS-12 score	Continuous variable	0.2858	0.982	0.951-1.015
Age	Continuous variable	0.5420	1.011	0.977-1.046
Associated diseases*	Yes vs no	0.6162	1.226	0.553-2.719
PCS-12 score	Continuous variable	0.6853	0.992	0.952-1.033
Rash area (lumbar dermatome)	Yes vs no	0.8261	1.111	0.435-2.836

*Presence of diabetes, chronic cardiovascular disease or chronic respiratory diseases.

Hosmer and Lemeshow goodness-of-fit test: p = 0.4098.

MCS-12 score: SF12 mental component summary score; PCS-12 score: SF12 physical component summary score.

The variables that were significantly predictive of pain present at 3 months were having more than 50 vesicles at V0, VAS score ≥3 at V0 and being male.

### Quality of life assessment

At the initial visit, the patients’ mean SF-12 scores were below the mean value of 50 (PCS-12 score = 40.1 ± 8.7, MCS-12 score = 41.7 ± 10.6 – Table 2). Both scores were lower in women and in older patients, particularly the PCS-12 score; patients without pain present at the visit had higher scores than those with pain. The mean PCS-12 and MCS scores in the study were lower than those in the healthy Italian population (Table 2) [34]. During the study, the PCS-12 and MCS-12 scores in patients presenting with pain at the respective visit did not vary, except for a small decrease in the MCS-12 score (Table 3).

## Discussion

The results from this prospective study, performed in a large network of GPs in Italy, show that patients aged ≥50 years report that HZ is painful, it has a negative impact on their QoL, working patients need to take sick leave and increasing age is a risk factor for severity. The incidence of PHN was high (20.6% at three-months and 9.2% at 6-months), although the majority of patients received antiviral treatment.

All the patients included had a rash even if the presence of rash was not an inclusion criterion, suggesting that the GPs diagnosed HZ based on the presence of the typical rash. We cannot exclude erroneous diagnosis since there was no laboratory confirmation. In the pivotal HZ vaccine clinical trial in adults, 5–6% of the clinical diagnoses were not confirmed in the laboratory [35]. Similarly, a prospective study of HZ diagnoses by GPs found 17% of diagnoses to be incorrect [36].

In HEROES, the patients enrolled do not seem to have suffered from severe disease as none were hospitalised. In Italy, the HZ hospitalization rate has been reported to be 5.6 per 100,000 patients and 10.3 per 100,000 for those with PHN [25]. The study was designed to have sufficient power to estimate the percentage of patients with HZ-associated pain, but was insufficient to detect HZ hospitalization. It is possible that patients with more severe disease may have consulted a specialist or were hospitalized directly. In addition, the low rate of consultations with specialists (12.3% during the six-month study) and the delay of about three days between rash onset and the GP visit suggest that the patients included in this study were suffering from non- severe to moderately-severe disease.

We observed a higher incidence of HZ-associated pain than that observed in the placebo group of the pivotal study (12% for PHN 3 months); however, the percentage of men was higher in the pivotal study and the subjects were older (≥60 years) so the age distribution was different [35]. In a French study, 11.6% of patients with HZ suffered from HZ-associated pain at 3-months, as declared by the patients whereas in a UK study there were about 27% of patients who suffered from PHN [16],[32]. These differences could be due to different access to healthcare and pain management or the method used to assess the HZ-associated pain. This last point is highlighted by the slightly different estimates for HZ-associated pain for the primary and secondary outcomes we observed in our study. Additionally, the VAS score was missing for 10.2% and 13.6% of patients at the 3- and 6-month visits, respectively; we cannot exclude that patients who suffered more from pain were more likely to complete the VAS score.

The percentage of patients with PHN reported in retrospective database studies ranged from 6.9% to 32% [21],[25],[33],[37]-[39]. Retrospective studies using International Classification of Disease (ICD) codes to extract data from databases do not always provide a reliable estimate of HZ and PHN incidences because of possible miscoding and under-coding. Additionally, the absence of a specific ICD code for PHN makes database extraction less reliable for this endpoint. The prospective design of our study, including three follow-up visits, enabled a better diagnosis of PHN than retrospective studies. Nevertheless, even if the actual estimates were different, the studies also generally reported an age-related increase in the percentage of patients with HZ-associated pain. The percentage of patients with PHN observed in HEROES was lower than the 40% assumed for the sample size estimation. Although we recruited fewer than the original target of 500 patients, we had data for the planned 400 patients needed to estimate the percentages of patients with PHN at 3 and 6 months with good precision. PHN can last for several months to several years but in this study we followed the patients for six months. This may have led to an underestimation of the true clinical burden of PHN. In this study, general practitioners recruited the participants. In Italy patients need to be referred for specialist care by their general practitioner, and even when they use private healthcare to consult a specialist directly, they have to report to their general practitioner for reimbursement of prescriptions via the national health system. Hence are results are likely to be generalizable in healthcare systems requiring referral for specialist appointments.

The presence of >50 vesicles, a VAS score ≥3 and being male were identified as variables that were associated with PHN at three months. The first two variables have been reported previously to be associated with a higher risk of developing PHN at three months suggesting that PHN is probably linked to more severe acute episodes of HZ [16]. However, other studies have either not reported any gender differences or have reported an increased risk in women, unlike our study. This may be due to differences in study methodology or, perhaps, in our study, women underestimated the level of their pain. It is also conceivable that there are more women in the older-age groups, so that there is confounding with age [40].

Most of the patients (91.5%) in HEROES received antivirals as recommended, which is similar to that reported in other studies [15],[16],[22]. In contrast, only 23% of our patients were reported as receiving pain therapy. This could be due to the low disease severity or because in Italy the most frequent pain therapies, i.e. paracetamol and NSAIDs are available without prescription and were, therefore, not reported by the physicians.

With the predicted ageing of the European population in the coming decades, the number of people affected by HZ and its related complications, including PHN, can also be expected to increase [41]. Preventive strategies, including vaccines, are thought to play an important role in the promotion of healthy ageing. Immunosenescence is reported to increase the risk of developing HZ due to an impaired T -cell-mediated immunity. In the Shingles Prevention Study, a live-attenuated Oka/Merck strain VZV vaccine was shown to reduce the incidences of HZ and PHN significantly, compared with placebo [35]. This vaccine is indicated in the EU for adults aged ≥50 years; in 2013, the UK started a shingles vaccination program targeting those aged between 70 and 79 years.

## Conclusions

These data suggest that there is an important burden of disease in the elderly due to HZ. There is a need for interventions that can prevent and reduce the burden of HZ to help improve the quality of life of the elderly. These data may be useful as baseline epidemiology data for the assessment of the impact of the VZV vaccine in Italy, after its implementation.

## Authors’ original submitted files for images

Below are the links to the authors’ original submitted files for images. Authors’ original file for figure 1 Authors’ original file for figure 2 Authors’ original file for figure 3

## Abbreviations

eCRF: Electronic case report form
EU: European Union
GP: General practitioner
HEROES: HERpes zoster Outcome: Epidemiological Study
HZ: Herpes zoster
ICD: International Classification of Disease
MCS-12: Mental component score
PCS-12: Physical component score
PHN: Post-herpetic neuralgi
QoL: Quality of life
SAS: Statistical Analysis System
SF-12: Short form-12 (quality of life questionnaire)
UK: United Kingdom
US: United States
VAS: Visual analogue scale
VZV: Varicella zoster virus
