# Supplementary figures and images for: Burden of herpes zoster-associated chronic pain in Italian patients aged 50 years and over (2009–2010): a GP-based prospective cohort study
Source: BMC Infect Dis. 2014 Dec 6;14:637. doi: 10.1186/s12879-014-0637-6 (PMC4268902; doi:10.1186/s12879-014-0637-6)

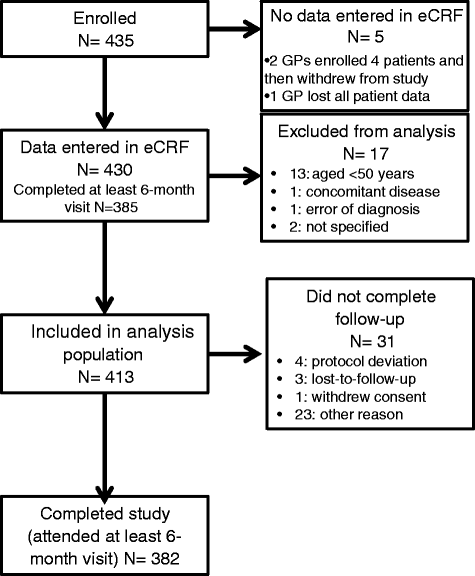

Supplement: Supplementary file 1 — Authors’ original file for figure 1 [file 12879_2014_637_MOESM1_ESM.gif]

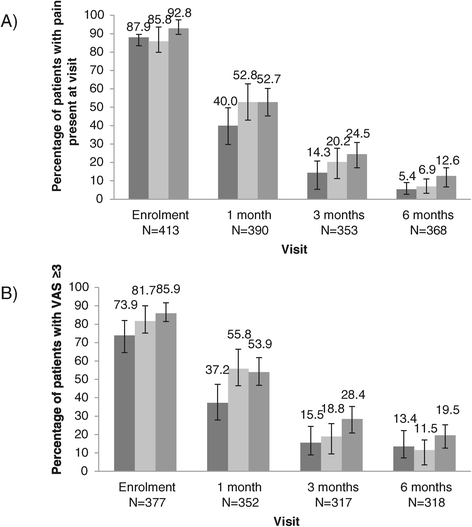

Supplement: Supplementary file 2 — Authors’ original file for figure 2 [file 12879_2014_637_MOESM2_ESM.gif]

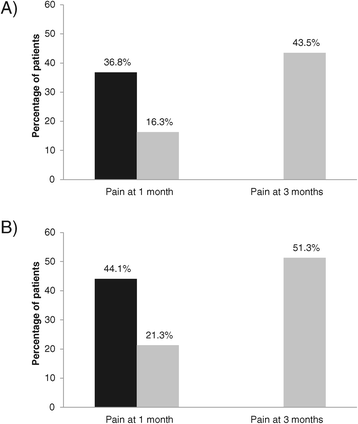

Supplement: Supplementary file 3 — Authors’ original file for figure 3 [file 12879_2014_637_MOESM3_ESM.gif]
